# Note on Fitting Trusses1Read at the thirty-third annual meeting of the Medical Association of Central New York, held at Rochester, N.Y., October 16, 1900.

**Published:** 1901-02

**Authors:** M. A. Veeder

**Affiliations:** Lyons, N. Y.


### NOTE ON FITTING TRUSSES.[1]

By M. A. VEEDER, M. D., Lyons, N. Y.

THE retention and much more the curability of hernias by the aid of trusses depend upon certain principles that require to be thoroughly understood and faithfully applied, in order to insure the best results.

The hernial canal, particularly when recently developed and not as yet much stretched, is, as a rule, oblique to the surface which it underlies, its outlet having the form of a more or less elongated slit, one lip of which commonly overlaps the other. This occurs in hernias

1. Read at the thirty-third annual meeting of the Medical Association of Central New York, held at Rochester, N.Y., October 16, 1900.

of every type and is a fact of prime importance in their treatment by trusses.

No matter what style of truss is employed, its pad requires to press on the hernial canal in such manner as to cause the overlapping lip at its outlet to be put on the stretch in the direction of the axis of the canal and outward from its lumen. When this is done the slit will be flattened and closed firmly and smoothly and the entire canal will likewise be in the same condition as far as it is possible to bring about such a result by the aid of a truss. If, on the other hand, the pressure of the pad tends to force the tissues in any other direction than that just described toward the outlet of the canal, the margins of the slit will be found puckered and wrinkled and stretched apart, preventing adhesion. Thus, in an ordinary direct hernia the writer has seen a truss so adjusted as to shove the overlapping margin at the orifice of its canal entirely off from that underlying it. The hernia was retained it is true, but in an uncomfortable and at times painful way, and without the slightest tendency toward cure. In fact the margins of the canal were plainly puckered and kept apart instead of being smoothed, the one upon the other and brought into accurate coaptation. A very little such pulling and pushing in the wrong direction prevents the formation of adhesions, facilitating retention and in favorable cases bringing about absolute cure.

Thus, a particular type of truss may have a fine effect in one case and fail utterly in another, the direction of pressure required in the two cases being entirely different even in hernias of the same class. Thus, an ordinary direct hernia may open obliquely through the muscles and fascia outward or inward in reference to the median line of the body, and the pressure of the pad of the truss should be directed either toward or away from that line, according to the special requirements of the case.

Proper management of the pressure on the skin and the subcutaneous fat and other tissues that are movable, upon the firmer underlying muscles and fascia in which the hernial canal properly so-called is situated, is likewise requisite. The skin and the loose tissues underlying it can readily be put upon the stretch by pulling them in any direction desired while under the pressure of the pad of the truss, which holds them quite firmly in whatever direction they may have been thus stretched. In this way it becomes possible to cause the superficial tissues in question to assist in the closure of the hernial canal by exerting a constant pull upon the overlapping margin of its outlet, to which they are more or less firmly attached. This disposal of these tissues under the pad is much more comfortable also than allowing them to be pressed upon and shoved in any other direction. The amount of traction thus brought to bear should be just enough to insure perfect coaptation, any excess beyond this being useless and perhaps disagreeable.

Simple as these points may seem they are of very serious importance. In almost any case if a truss is carefully adjusted in accordance with the principles that have been indicated, and if this is always done before assuming the upright position, so as never to allow the hernia to protrude for an instant, ease of retention will be greatly increased in all cases, and the percentage of cures become very much larger. The length of time required varies according to the nature of each case, but improvement will continue for two or three years, even if a cure has not resulted in that interval of time.

From the point of view indicated it is necessary to study carefully the direction of pressure exerted by trusses of different types, so as to be able to select just what is needed for each particular case. It is needful also to have the patient return repeatedly, so that it may be seen whether every detail is properly understood. Patients who have little mechanical skill, or who are careless need to be drilled. Sometimes also it becomes necessary to adjust pads to make the counter pressure less uncomfortable and to modify the action of the truss slightly. Such pads for counter pressure may be made and adjusted by any harness-maker and a little ingenuity in their application is often of great service. In any event there can be no very decided success in the use of trusses if the points here stated are ignored.

